# Design and Synthesis of Hybrid Compounds as Epigenetic Modifiers

**DOI:** 10.3390/ph14121308

**Published:** 2021-12-15

**Authors:** Juliana Romano Lopes, Igor Muccilo Prokopczyk, Max Gerlack, Chung Man Chin, Jean Leandro Dos Santos

**Affiliations:** School of Pharmaceutical Sciences, São Paulo State University (UNESP), Araraquara 14800-903, Brazil; jromanolopes@gmail.com (J.R.L.); improko@gmail.com (I.M.P.); maxgerlack96@hotmail.com (M.G.); chung.man-chin@unesp.br (C.M.C.)

**Keywords:** histone deacetylase, bromodomain, epigenetic, polypharmacology, hybrid, drug design, synthesis, molecular hybridization

## Abstract

Epigenetic modifiers acting through polypharmacology mechanisms are promising compounds with which to treat several infectious diseases. Histone deacetylase (HDAC) enzymes, mainly class I, and extra-terminal bromodomains (BET) are involved in viral replication and the host response. In the present study, 10 compounds were designed, assisted by molecular docking, to act against HDAC class I and bromodomain-4 (BRD4). All the compounds were synthesized and characterized by analytical methods. Enzymatic assays were performed using HDAC-1, -4, and -11 and BRD4. Compounds (**2**–**10**) inhibited both HDAC class I, mainly HDAC-1 and -2, and reduced BRD4 activity. For HDAC-1, the inhibitory effect ranged from 8 to 95%, and for HDAC-2, these values ranged from 10 to 91%. Compounds (**2**–**10**) decreased the BRD4 activity by up to 25%. The multi-target effects of these compounds show desirable properties that could help to combat viral infections by acting through epigenetic mechanisms.

## 1. Introduction

Polypharmacology is a drug design approach that has been investigated to address efficacy and safety issues for diseases in which there is an involvement of multifactorial pathways, such as those found for several infectious diseases [1,2]. This approach aims to disrupt biological networks through interference with multiple pathways, providing potent and robust responses for treatment, avoiding drug–drug interactions, minimizing drug resistance, and providing simplified therapeutic schemes [2]. The multi-target drugs (hybrid drugs) obtained from this approach can be explored to design new compounds acting through dual epigenetic mechanisms [3].

Epigenetic modifiers control the expression or silencing of genes in the so-called “epigenetic code”. The basic unit of chromatin, histones, can undergo remodeling processes through different enzymes such as: (a) “writers”, capable of making covalent modifications in the histones, as the histone acetyltransferases (HATs) (b) “erasers”, responsible for removing the covalent changes made by the “writers”, as the histone deacetylases (HDACs), and (c) “readers”, proteins able to recognize post-translational modifications in the histones without causing any changes in their structure, for example, the recognition of *ε-N*-acetylation of lysine residues by bromodomains (BRD) [4].

HDAC enzymes, characterized as “erasers”, act by removing the acetyl groups of lysine residues on the histone tail, hampering the access to DNA by the transcription machinery, leading to a reduction in gene expression. HDACs are divided into groups based on their homology and are classified into: class I (HDAC 1, 2, 3, and 8), class II (HDAC 4, 5, 6, 7, 9, and 10), class III (SIRT1–7), and class IV (HDAC 11). These classes differ essentially for being zinc-dependent (HDAC class I, II, and IV) or NAD-dependent (HDAC class III) [5]. Among the HDAC actions, the regulation of viral transcription, viral budding and trafficking, enhancement of endocytosis, and uncoating have been described [3,4,5,6,7,8]. Thus, the role of HDAC enzymes in the replication of numerous viruses has already been described for HIV-1, hepatitis (i.e., HBV, HCV), respiratory syncytial virus (HSV), human papillomavirus (HPV), *Herpesviridae* family members (i.e., herpes simplex virus (HSV), human cytomegalovirus (HCMV), and Epstein–Barr virus (EBV)) [6,7,8]. Among HDAC classes, it seems that those belonging to class I, mainly HDAC-1, -2, and -3, have important roles against DNA and RNA viruses [8]. HDAC inhibitors have also been investigated for integrative retroviruses (HIV and human T-cell leukemia virus type 1, HTLV-1) infections, as latency reversal agents (LRA), exploring the ‘Kick and Kill’ approach [9,10,11]. According to this strategy, also known as ‘shock and kill’, the latent viral reservoir could, in theory, be purged in two steps. Firstly, the LRA could stimulate viral gene expression and bud from latent CD4+ cells and subsequently, the immune system recognizing those infected cells would activate T-lymphocyte cell-induced toxicity or even act through a viral cytopathic effect [12]. For HIV, such an approach has demonstrated promising in vitro and in vivo results; however, challenges related to the measurement and accurate characterization of the reservoirs demand additional efforts for the use of HDAC inhibitors (HDACi) as LRA in therapies. Indeed, some studies suggest that combined treatment using other LRA classes, such as bromodomain inhibitors, could synergically activate latent viral reservoirs in a more robust way [13,14].

On the other hand, bromodomains (BRD) are protein modules evolutionarily conserved, containing about 110 amino acids, and are able to recognize ε-*N*-acetylated lysine residues, such as those present in histone tails or chromatin-associated proteins. BRD are grouped into eight families of 46 different proteins [15,16]. Bromodomain inhibitors have been investigated in clinical trials for oncology and non-oncology (i.e., type 2 diabetes mellitus, cardiovascular diseases, coronary artery diseases, chronic kidney failure, among others) indications [16]. The family II extra-terminal bromodomains (BET) (BRD2, BRD3, BRD4, and BRDT) are also involved in several viral infections since the replication and transcriptional viral regulation depends on BRD recognition. BRD and BET inhibitors block two distinct steps in the EBV lytic cycle by inhibiting the viral protein BZLF1 and downregulating the host protein BACH1, hampering viral gene expression. JQ1, a BET inhibitor, also reduces BRD4 recruitment for viral replication [17]. Experiments using Calu-3 cells revealed that the BET inhibitor apabetalone (RVX-208) attenuates the severe acute respiratory syndrome coronavirus 2 (SARS-CoV-2) infection by downregulating the expression of cell surface receptors such as angiotensin-converting enzyme 2 (ACE2) and dipeptidyl-peptidase 4 (DPP4 or CD26) [18]. BRD4 binds to HPV protein E2, inhibiting the recruitment of the positive transcription elongation factor (PTEFB) to viral chromatin, repressing the viral oncogenes E6 and E7 [19,20]. Curiously, inhibitors such as JQ1, improve HPV 16 DNA replication [19]. For HIV, BRD inhibitors can activate latent reservoirs, since BRD4 has been described to compete with HIV Tat protein for PTEFB binding [21,22].

**Figure 1:**
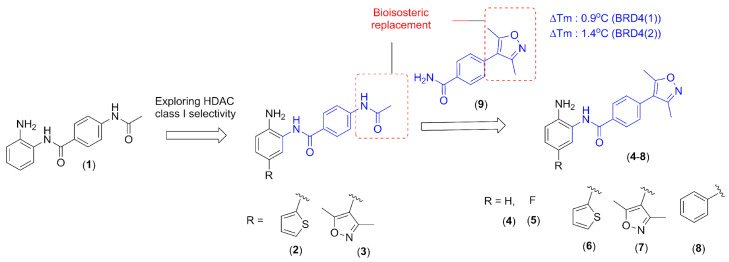
Drug design of the novel epigenetic modifiers.

In this context, the design of hybrid compounds acting through dual bromodomain (BRD4) and HDAC class I inhibitors seems to be a promising approach for several viral infections. Here, the chemical structure of **1** (CI-994, tacelidine) was modified aiming to improve the selectivity for HDAC class I (Figure 1) and to gain an additional BRD4 inhibitory effect. Tacelidine (**1**) was used as a prototype due to its effect against HDAC class I. This drug exhibited IC_50_ values for human HDAC-1, HDAC-2, and HDAC-3 of 0.9 µM, 0.9 µM, and 1.2 µM, respectively [5,6,7,8]. The selectivity for HDAC-1 and HDAC-2 inhibition could be improved by the inclusion of heteroaromatics that occupies the additional 14 Å pocket present in both enzymes [5,6,7,8,10]. Here, we investigated the effect of thiophene (**2**) and 3,5-dimethyloxazole (**3**) insertion in the prototype (**1**) to improve the inhibition of HDAC-1 and -2. Such selectivity seems to be important for HBV, HSV-1, EBV, HPV, HIV, and HSV viral infections [5,6,7,8,9,10].

For BRD4, structural data revealed that the recognition of acetylated lysine occurs in the hydrophobic pocket, in which an asparagine residue was found [23]. Among the acetyl-lysine mimetics, 3,5-dimethyl-isoxazole subunit was used to design ligands against BET-bromodomains, since it shows good ligand efficiency and selectivity [24]. Previously, our research group identified the 3,5-dimethylisoxazole fragment (**9**) using differential scanning fluorimetry (DSF) as a BRD4 inhibitor with ∆T_m_ (°C) values of 0.9 °C (BRD4(1)) and 1.4 °C (BRD4(2)) at 20 μM

(results not published). Exploring the bioisosteric relationship between an acetamide subunit present in the tacelidine (**1**) and 3,5-dimethyl-isoxazole subunit in the fragment (**9**), a new series of 2-amino-benzamide derivatives were designed, aiming to target both BRD4 and HDAC class I (Figure 1).

## 2. Results and Discussion

The design of dual inhibitors HDAC/BRD has been explored in recent years, mainly for cancer treatment [25]. Several studies have shown the efficacy of these hybrid compounds to induce apoptosis and growth arrest in different types of tumor cells, and its efficacy has been evaluated in clinical trials [26]. The rationale for structural-based designs of dual HDAC/BRD inhibitors has been published elsewhere [27]. Previously, dual BRD4/HDAC inhibitors containing dimethylisoxazoles showed anti-proliferative effects on chronic myeloid leukemia (CML) and AML cell lines [28]; however, for these compounds, the hydroxamic acid subunit did not provide selective against the HDAC family. In addition, those compounds were not evaluated against viral infectious diseases. In this work, we explored—via molecular modifications—the design of epigenetic modifiers acting against HDAC class I and BRD4. The mode of binding was studied using molecular modeling and all compounds were synthesized and evaluated through enzymatic assays using HDAC-1, -2, -3, and BRD4.

### 2.1. Molecular Docking and In Silico ADME Properties

In general, molecular docking procedures were performed to suggest possible modes of interactions and the occupancy of HDAC-2 and 3 and BRD4 by the designed compounds (1–10) for this study. It was expected that the 2-aminobenzamide moiety was oriented towards Zn^+2^ in the active site for HDAC-2 and HDAC-3. The acetamide group was expected to be outside the active site for HDAC-2 and -3, while for BRD4, this subunit could interact with ASN140 or conserved water molecules inside the pocket. Table 1 shows the docking score (DS) values for HDAC-2, HDAC-3, and BRD4. For HDAC-2, the DS values ranged from −6.940 to −11.101, while for HDAC−3, these values ranged from −7.548 to −8.490. For BRD4, it was found the DS values ranged from −2.956 to 6.014. 

**Figure 2:**
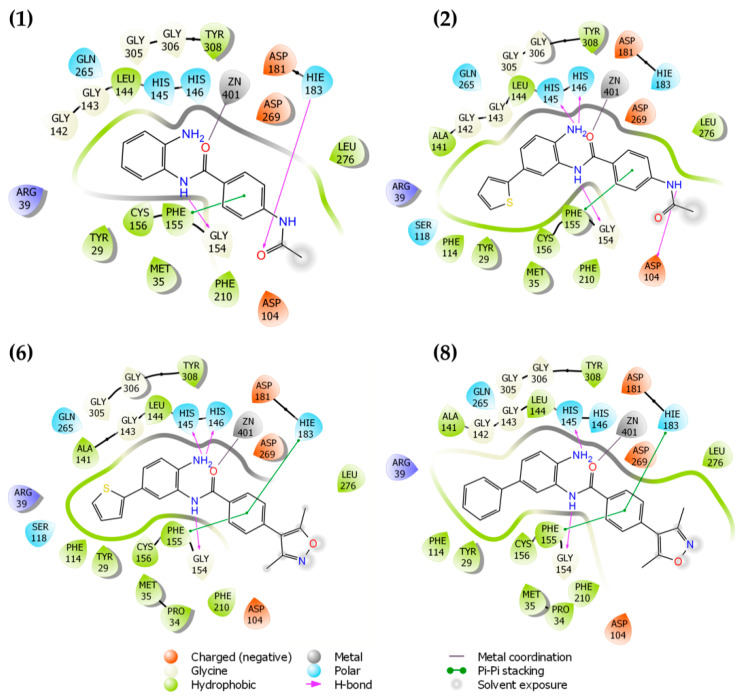
Two-dimensional (2D) representation of ligand interaction diagram for compounds **1**, **2**, **6**, and **8** docked on HDAC-2.

Figure 2 shows the possible binding mode of the ligands against HDAC-2. It is possible to observe the same patterns of interaction mode for the ligands. The presence of the thiophene ring (**2**,**6**) or phenyl (**8**) ring occupies the cavity of 14 Å near Zn^+2^, exhibiting similar binding modes. In comparison with compound (**1**), in which this pattern of replacement does not exist, it is possible to visualize a reduction in the DS values. Compounds **1** and **2** interact with HIS183 and ASP104 residues, respectively. For compounds **6** and **8**, these interactions are absent since the compounds present 3,5-dimethylisoxazole instead of the acetamide subunit. The docking studies for HDAC-2 inhibitors proved to be consistent with enzymatic results, as shown in Section 3.3.

**Figure 3:**
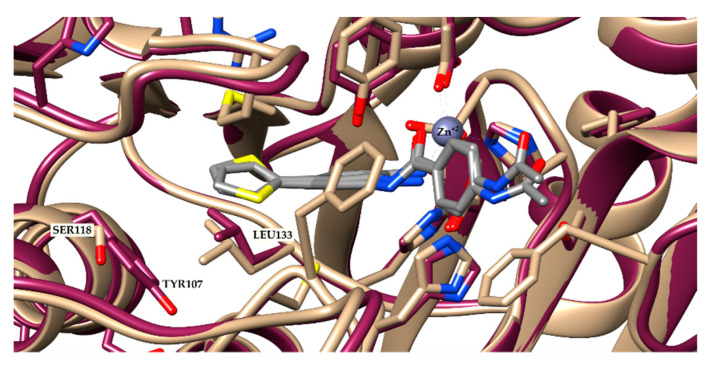
Superposition of HDAC-2 (beige) and 3 (purple). In grey—a co-crystallized ligand from HDAC-2 (PDB ID: 4LY1).

On the other hand, HDAC-3 is not found in the 14 Å cavity near Zn^+2^. The TYR107 in HDAC-3 pushes LEU133, hampering access to this cavity (Figure 3). This modification renders HDAC-3 unable to fit ligands with voluminous groups. Indeed, the docking results confirmed this observation, since only four compounds had poses recovered by the docking procedure, and all showed the acetamide moiety oriented towards Zn^+2^.

**Figure 4:**
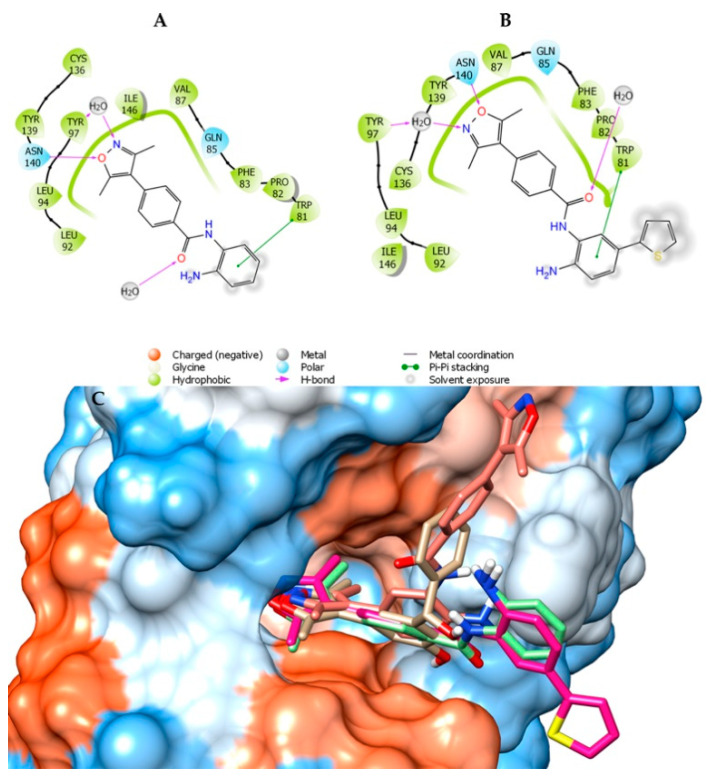
Ligand interaction diagram of compounds **4** (**A**), **6** (**B**), and **7** (**C**). Superposition of compounds **4** (light green), **6** (magenta), and **7** (salmon) with **1H3** (beige) co-crystallized with BRD4 (PDB ID: 4J0S).

The 3,5-dimethylizoxazole group acts as a lysine mimetic in the active site of BRD4 [29]. Figure 4 shows a comparison of three designed compounds (**4**, **6**, and **7**) with the BRD4 ligand named 1H3 (PDB code: 4J0S). Compounds (**4**, **6**, and **7**) exhibited high similarity (position and occupancy) compared to 3,5-dimethylizoxazole. The difference between **4** (Figure 4A) and **6** (Figure 4B) is the inclusion of a thiophene ring (**6**) that, by docking, appears not to be favorable, since the thiophene ring was exposed. Compound **7,** containing two 3,5-dimethylizoxazole subunits, maintains a different orientation compared to thiophene substitution (Figure 4C).

In silico ADME properties were obtained using the SwissADME tool [30,31] (Supplementary Material—Table S1). Physicochemical properties (clogP, water solubility), pharmacokinetic parameters (gastrointestinal absorption, blood-brain barriers permeation, CYP inhibition), and drug likeness (according to all Lipinski, Ghose, Veber, Egan or Muegge descriptors) were characterized in order to evaluate the most promising compounds to be further evaluated in vitro and in vivo. cLogP values ranged from 1.70 to 3.26 and compounds **2**–**10** were more lipophilic than tacelidine (**1**). Compounds **2**–**10** were characterized as water soluble (**4**,**10**) and moderately soluble (2, 3, and 5–9). Except for (**6**), all compounds exhibited high gastrointestinal absorption. In addition, compounds **1**–**10** exhibited drug-likeness properties in accordance with oral absorption descriptors. For those compounds containing nitro (**9**,**10**), a structural alert regarding possible genotoxicity was found.

### 2.2. Chemistry

**Figure 5:**
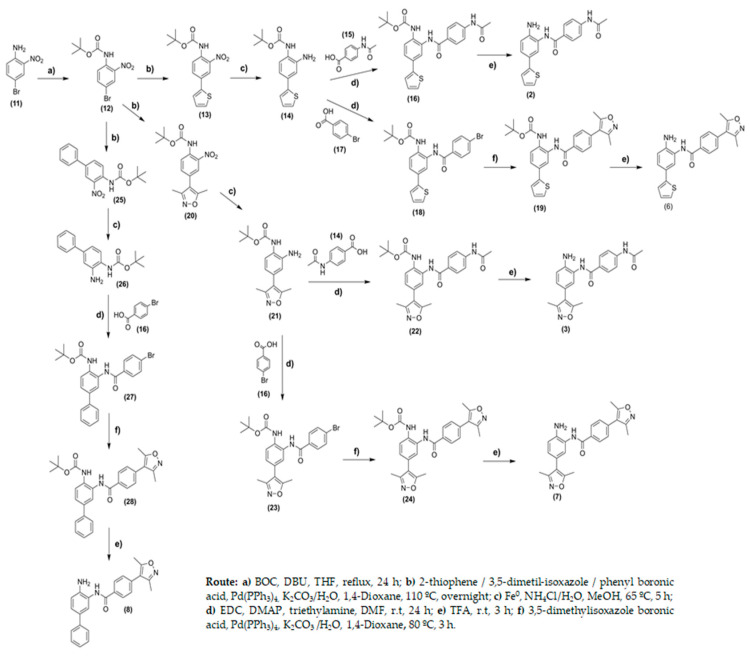
Synthesis of compounds (**2**), (**3**), (**6**), (**7**), and (**8**).

**Figure 6:**
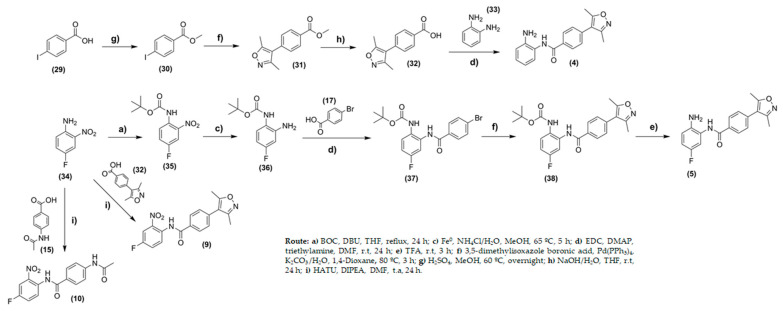
Synthesis of compounds (**4**), (**5**), (**9**), and (**10**).

The synthetic routes for the synthesis of compounds (**2**–**10**) are outlined in Figure 5 and Figure 6. Initially, the protection of the amino group in 4-bromo-2-nitro-aniline (**11**) was realized using di-tert-butyl dicarbonate in a medium containing THF as a solvent and 1,8-diazabicyclo [5.4.0]undec-7-ene (DBU) as a base. After purification, compound (**12**) was obtained at yields of 29%. Compounds (**13**), (**20**), and (**25**) were obtained through Suzuki coupling reactions using the appropriate boronic acid derivatives and Pd(PPh_3_)_4_, as a catalyst, at yields ranging from 59 to 73% (Figure 5). The reduction in nitro groups using iron and ammonium chloride in water/ethanol medium yielded the amine intermediates (**14**), (**21**), and (**26**) at yields ranging from 45 to 72%. From common intermediates (**14**), (**21**), and (**26**), it was possible to synthesize compounds (**2**–**8**) exploring two or three additional steps. Compounds (**16**), (**18**), (**22**), and (**23**) were obtained through coupling reactions involving the amine derivatives (**14**), (**21**), and (**26**) and 4-acetamide-benzoic acid or 4-bromo-benzoic (**17**) acid using *N*′-ethylcarbodiimide hydrochloride (EDC), 4-(dimethylamino)pyridine (DMAP) as coupling reactions in the medium containing anhydrous *N*,*N*-dimethylformamide. For these compounds, the yields ranged from 13 to 29%. Compound (**16**) was previously converted to acyl chlorides using oxalyl chloride in dichloromethane medium to provide compound (**27**) at a yield of 84%.

For compounds (**18**), (**23**), and (**27**), Suzuki coupling reactions were carried out using 3,5-dimethyl-isoxazole boronic acid in a 1,4-dioxane medium at yields of 35%. For compounds (**16**), (**19**), (**22**), and (**24**) the Boc-protecting group was removed using trifluoroacetic acid to provide compounds (**2**), (**3**), (**6**), (**7**), and (**8**) at yields ranging from 67 to 88% (Figure 5). Similar procedures were conducted for the synthesis of compound (**5**), however, the starting material, in this case, was 4-fluoro-2-nitro-aniline (**34**). Compound (**5**) was obtained in five steps at global yields of 4.13% (Figure 5).

Compound (**4**) was prepared through four steps (Figure 6). First, iodo-benzoic acid was converted to the ester (**30**) to protect the carboxylic acid. In the second step, the Suzuki coupling reaction was carried out by treatment of (**30**) with 3,5-dimethyl-isoxazole boronic acid and Pd(PPh_3_)_4_, as a catalyst, using 1,4-dioxane as a solvent. Compound (**31**) was obtained at yields of 64%. The ester group was hydrolyzed in basic medium leading to (**32**). In the last step, ortho-phenylenediamine (**33**) was treated with (**32**) using *N*′-ethylcarbodiimide hydrochloride (EDC) and 4-(dimethylamino)pyridine (DMAP) in *N*,*N*-dimethylformamide to provide compound (**4**) with a yield of 15%.

Both 4-fluoro-2-nitro-benzamides (**9** and **10**) were obtained from the coupling reactions with 4-(3,5-dimethylisoxazol-4-yl)benzoic acid (**32**) or 4-acetamidobenzoic acid (**15**), respectively. The reactions used 1-[bis(dimethylamino)methylene]-1H-1,2,3-triazolo[4,5-b]pyridinium 3-oxide hexafluorophosphate (HATU) as a coupling reagent, and the yields were 55% (**9**) and 26% (**10**) (Figure 6). Analytical methods, including infrared (IR) spectroscopy, mass spectrometry, and ^1^H and ^13^C nuclear magnetic resonance (NMR) spectroscopy, were used to characterize the chemical structures. For all compounds (**1**–**10**), the purity, analyzed by high-performance liquid chromatography (HPLC), was shown to be superior to 98.7%.

### 2.3. Enzymatic Inhibition Assays

Compounds (**1**–**10**) were evaluated at 10 µM against HDAC-1, -2, -3, -4, -6, -11, and BRD4 (Table 2). The inhibitory effect ranged from 8 to 95% for HDAC-1, 10–91% for HDAC-2, and 3–77% for HDAC-3. Compounds (**2**–**10**) were not active against HDAC-4 and HDAC-11. On the other hand, for BRD4, these values ranged from 0 to 25%. Vorinostat, a pan-HDAC inhibitor, exhibited a percentage inhibition of about 90% at 0.5 µM, being inactive against BRD4. JQ-1, a BET-bromodomain inhibitor, reduced the BRD4 activity by 88% at 2.5 µM.

Compounds (**2**–**8**) were more prone to inhibit HDAC-1 and HDAC-2 than other isoforms. Compounds (**2**) and (**6**) were the most active to inhibit HDAC-1, while for HDAC-2, compound (**2**) exhibited a more pronounced effect by inhibiting 91% of the enzyme activity. Compound (**4**) was the most active against HDAC-3. 

Compounds containing thiophene (**2**) and 3,5-dimethylisoxazole (**3**) substitutions were designed to occupy the 14 Å hydrophobic pocket present in HDAC-1 and -2. Based on the results, the presence of thiophene substitution favors the inhibitory effect of HDAC-1 and -2 over HDAC-3. Similar results were found for compound (**6**), in which the percentage inhibition for HDAC-1, 2, and 3 was 95%, 88%, and 16%. For compound (**8**), in which the phenyl ring is found in this position, values of inhibition for HDAC-1 and -2 were 92% and 76%, respectively. The presence of phenyl in compound (**8**) seems to confer selectivity for these HDAC isoforms. In addition, 3,5-dimethylisoxazole substitution present in compounds (**3**) and (**7**) at the same position was not favorable since values of HDAC inhibition were ≤10% for all isoforms. However, for BRD4, both compounds (**3**) and (**7**) were the most active, reducing the BRD4 activity by 25%. These data suggest that the 3,5-dimethylisoxazole group is sterically unfavorable to be adjusted in the 14 Å pocket. In addition, the presence of the 2-aminobenzamide subunit was confirmed to be essential for the effect, since compounds (**9**) and (**10**) exhibited weak activity. Due to the lack of activity against HDAC, these compounds were not evaluated against BRD4. 

The bioisosteric replacement of hydrogen (**1**) by fluorine (**5**) reduces the activity against HDAC 1–3. Despite this unfavorable effect on HDAC, compound (**5**) also acted on BRD4, by decreasing about 20% of the activity. Compound (**1**) did not exhibit an effect on BRD4. The results found for BRD4 revealed a moderate effect of these compounds against this target. Moreover, the bioisosteric replacement of acetamide by 3,5-dimethylisoxazole (Figure 1) maintains the molecular recognition of BRD4, probably due to its acetyl-lysine mimetic effect. All compounds (**2**–**8**) exhibited effects acting against BRD4 and HDAC class I, mainly HDAC-1 and -2.

Interestingly, the potential of compound (**2)** to activate latent HIV-1 in U1 cells has been previously demonstrated [32]. In these cells, compound (**2**) was more active than vorinostat, exhibiting an EC50 value—measured by the levels of viral p24 produced—of 0.93 µM [32]. These data suggest the potential of epigenetic modifiers acting against BRD4 and HDAC class I for viral infectious diseases.

## 3. Materials and Methods

### 3.1. Computational Methods

#### 3.1.1. Molecular Docking

In silico studies were carried out on the Schrodinger (2019-4) Maestro v12.2 molecular modeling environment using a computer containing an Intel Core I7-4790 processor with 16 Gb memory and a Nvidia GeForce GTX 980 graphic processor. For this study, targets used included: BRD-4 (PDB ID: 4WIV; resolution: 1.56 Å) [33,34], HDAC-2 (PDB ID: 4LY1; resolution: 1.57 Å) [35], and HDAC-3 (PDB ID: 4A69; resolution: 2.06 Å), which were retrieved from Protein Data Bank (PDB). The referred structures were crystallized with their respective inhibitors: 3P2 for BRD-4 and 20Y (compound **2**) for HDAC-2 [34,35]. Since protein structure is not directly suitable for the use of molecular modeling [29], protein preparation was conducted on *Protein Preparation Wizard* as follows: (i) removal of water molecules (except those preserved in the active site for BRD-4); (ii) adding hydrogen atoms; (iii) filling in incomplete side chains; (iv) energy minimization using OPLS3 force field. All residues were optimized based on pH 7.0 ± 2.0. The minimization steps were performed up to threshold RMSD values equal to 0.15 Å. The interaction box (grid) was defined by the Grid Generation Receiver with the dimensions: 10 Å × 10 Å × 10 Å. The 2D ligands library was imported into Maestro^®^ and prepared using Ligand Preparation (LigPrep) to optimize 3D geometry using an OPLS_2005 force field and EPIK to generate possible protonation states at pH 7.00 ± 2.00 [36]. Tautomers and stereoisomers were assessed, and for docking analysis, 20 poses per ligand were generated. Other parameters were kept as default. Extra-precision mode (XP) was used for docking simulations [37].

#### 3.1.2. In Silico Prediction of ADME Properties

Theoretical studies were performed to determine the potential of oral absorption, drug likeness, and water solubility of the compounds (**1**–**10**) using the Swiss ADME software [30,31].

### 3.2. Chemistry

#### 3.2.1. General

Solvents and reagents were purchased from commercial suppliers and for reactions all solvents were dried before use. The reactions were monitored using thin-layer chromatography (TLC), precoated with silica gel 60 (HF-254; Sigma-Aldrich, St. Loius, MA, USA) to a thickness of 0.25 mm. The plates were revealed under UV light (254 nm) and, when necessary, treated with ninhydrin to detect primary amines. All compounds were purified on a chromatography column with silica gel (60 Å pore size, 35–75 µM particle size) using appropriate mobile phases, as described for each compound. The purity for all compounds was characterized by HPLC using a Shimadzu LC-10AD chromatograph equipped with a model SPD-10A UV–vis detector (Shimadzu, Kyoto, Japan). For this study, all compounds exhibited purity superior to 98.5%. Melting points (mp) were determined in open capillary tubes using an electrothermal melting point apparatus (SMP3; Bibby Stuart Scientific, Stone, UK). Infrared (IR) spectroscopy (KBr disc) was performed on an FTIR-8300 Shimadzu spectrometer (Shimadzu, Kyoto, Japan), and the frequencies were expressed per cm^−1^. Nuclear magnetic resonance (NMR) spectra for ^1^H and ^13^C of all compounds were obtained on a Bruker DRX-600 (600 MHz) NMR spectrometer (Billerica, MA, USA) using deuterated solvents for sample preparation. Chemical shifts were expressed in parts per million (ppm) relative to tetramethylsilane. The coupling constants were reported in hertz (Hz), and the signal multiplicities were reported as singlet (s), doublet (d), doublet of doublet (dd), doublet of doublet of doublets (ddd), triplet (t), and multiplet (m). Mass spectra were acquired using an LC-DAD-ESI system from Shimadzu HPLC (CBM20A) (Shimadzu, Kyoto, Japan), LC-20AD quaternary pump, SPD-M20A detector, SIL-20A autosampler, and CTO-20A column compartment, coupled to a Bruker Ion Trap ESI source (amaZon SL). Mass analysis was performed in positive mode and m/z scanned 50–1000 using the following parameters: source voltage of 4.5 kV, 9.00 L/min sheath gas, 40 psi nebulizer and dry temperature (300 °C). Compounds **1** (CI-994, tacelidine) [38], **12** [39], **13** [39], **14** [39], **25** [40], **26** [40], and **35** [41] were prepared according to a previously described methodology.

#### 3.2.2. Synthesis of Tert-butyl (4-(3,5-dimethylisoxazol-4-yl)-2-nitrophenyl)carbamate (**20**)

In a 10 mL round-bottom flask, 0.6 mmol of tert-butyl (4-bromo-2-nitrophenyl) carbamate (**12**), 0.78 mmol of the boronic acid derivative, 0.03 mmol of Pd(PPh_3_)_4_ (palladium tetrakis), 1.2 mmol of K_2_CO_3_ in 2 mL of water, and 5 mL of 1,4-dioxane were added. The Suzuki coupling reactions were carried out at 100 °C for 18 h under a nitrogen environment. The reaction was monitored by TLC using hexane:ethyl acetate (6:4) as the mobile phase. For isolation, the medium was cooled and filtered through celite to remove the palladium catalyst, washed with MeOH, and the solvent was dried leading to a brown crude. This solid was suspended in ethyl acetate (150 mL) and the organic phase was washed three times within 50 mL of distilled water. Subsequently, the organic phase was dried over anhydrous sodium sulfate and filtered. Then, the solvent was removed under reduced pressure and the compound was purified by chromatography to obtain the desired product (**20**).

The compound tert-butyl (4-(3,5-dimethylisoxazol-4-yl)-2-nitrophenyl)carbamate (**20**) was purified by chromatography using the Biotage^®^ Isolera TM (SNAP ultra 10; with hexane/ethyl acetate 7:3 as eluents in an isocratic method; 36 mL/min flux and 254 nm wavelength detection), leading to the formation of the pure product, as a brown solid at yield of 62%. **^1^**H NMR (600 mHz, DMSO- *d*_6_) δ 9.70 (s, 1H); 7.92 (m, 1H); 7.70 (m, 2H); 2.42 (s, 3H); 2.24 (s, 3H); 1.45 (*s*, 9H); ^13^C (150 mHz, DMSO- *d*_6_) δ 11.32, 158.11, 10.35, 152.47, 125.72, 131.60, 134.80, 114.01, 125.08, 124.50, 141.47, 165.96, 80.63, 27.90.

#### 3.2.3. General Procedures for the Synthesis of Tert-butyl (2-amino-4-phenyl)carbamate (**21**), (**26**) and (**36**)

In a 10 mL round bottom flask, 0.6 mmol of the compound (**13**) or (**20**) or (**25**) or (**35**) was added with 2.4 mmol of Fe(0), 3 mmol of NH_4_Cl in a mixture of solvents containing 2 mL of H_2_O and 7 mL of methanol. The reaction was carried out at a temperature of 60 °C and stirred for 5 h. For monitoring the reaction by TLC, hexane:ethyl acetate (1:1) was used as the mobile phase. Next, the reaction medium was filtered through celite to remove the iron, washed using methanol, and evaporated to remove the solvent leading to a brown crude. This solid was suspended in ethyl acetate (150 mL) and the organic phase was washed three times with 50 mL of distilled water. Subsequently, the organic phase was dried over anhydrous sodium sulfate and filtered. Then, the solvent was removed under reduced pressure and the compound was purified by chromatography to obtain the desired products (**21**) and (**36)**

##### Tert-butyl (2-amino-4-(3,5-dimethylisoxazol-4-yl)phenyl)carbamate (**21**) 

Compound tert-butyl (2-amino-4-(3,5-dimethylisoxazol-4-yl)phenyl)carbamate **(21)** was purified by chromatography using Biotage^®^ Isolera TM (SNAP ultra 10; with hexane/ethyl acetate 7:3 as eluents in an isocratic method; 36 mL/min flux and 254 nm wavelength detection) leading to the formation of the pure product, a light brown solid at yield of 45%. **^1^**H NMR (600 mHz, DMSO- *d*_6_) δ 8.38 (s, 1H); 7.28 (d, 1H); 6.68 (d, 1H); 6.51 (dd, 1H); 4.97 (s, 2H); 2.37 (s, 3H); 2.19 (s, 3H); 1.47 (s, 9H); ^13^C (150 mHz, DMSO- *d*_6_) δ 11.35, 158.11, 10.58, 152.47, 126.15, 123.17, 115.85, 116.07, 116.84, 124.56, 141.30, 164.44, 28.17, 78.85. 

##### Tert-butyl (2-amino-4-fluorophenyl)carbamate (**36**)

The compound tert-butyl (2-amino-4-fluorophenyl)carbamate (**36**) was purified by chromatography using Biotage^®^ Isolera TM (SNAP ultra 10; with hexane/ethyl acetate 6:4 as eluents in an isocratic method; 36 mL/min flux and 254 nm wavelength detection), leading to the formation of the pure product, as a yellow solid at a yield of 86%. **^1^**H NMR (600 mHz, DMSO- *d*_6_) δ 8.41 (s, 1H); 7.19 (m, 1H); 6.65 (dd; *J =* 6.9, 1.5 Hz; 2H); 4.77 (s, 2H); 1.46 (s, 9H).

#### 3.2.4. General Procedures for the Synthesis of 4-acetyl-*N*-(2-amino-5-phenyl)benzamide derivatives (**2**) and (**3**)

In a 10 mL round-bottom flask, 1.6 mmol of 4-acetamidobenzoic acid (**15**) was added 1.6 mmol of *N*′-ethylcarbodiimide hydrochloride (EDC), 0.3 mmol of 4-(dimethylamino)pyridine (DMAP), and 5 mL of anhydrous *N*,*N*-dimethylformamide (DMF). The reaction was kept under constant stirring and nitrogen atmosphere for 30 min. Next, about 1.6 mmol of tert-butyl (2-amino-4-phenyl)carbamate (**13**) or (**17**) was added dropwise at 0 °C and the reaction was maintained under stirring at room temperature for 24 h. For monitoring the reaction by TLC, hexane:ethyl acetate (1:1) was used as the mobile phase. Then, the medium was diluted with 150 mL of ethyl acetate and the organic phase was washed three times with 50 mL of distilled water. The organic phase was dried with anhydrous sodium sulfate and filtered. Then, the solvent was evaporated under reduced pressure and the compound was purified by chromatography to obtain the Boc-protected intermediates (**16**) or (**22**) with 13 and 14% yields. The last step consisted of the removal of the Boc-protecting group, by the addition of about 0.2 mmol of the respective previous intermediate and trifluoroacetic acid (TFA) in excess (1.1 mmol) to a 10 mL round-bottom flask. The reaction was maintained at room temperature and stirred for 3 h. The reaction was monitored by TLC, using the ethyl acetate:hexane (1:1) elution as the mobile phase, developed under ultraviolet light (254 nm). Ninhydin (2,2-dihydroxy-hydrindene-1,3-dione) solution at 5% was used for the detection of primary amines. The TFA was removed under reduced pressure, and the solid obtained was suspended with 150 mL of ethyl acetate. The organic phase was washed three times with 50 mL of distilled water. The organic phase was dried over anhydrous sodium sulfate and filtered. Lastly, the solvent was evaporated under reduced pressure to obtain the desired products (**2**) or (**3**).

##### 4-Acetyl-*N*-(2-amino-5-(thiophen-2-yl)phenyl)benzamide (**2**)

Brown solid with 88% yield. **^1^**H NMR (600 mHz, DMSO- *d*_6_) δ 10.22 (s, 1H); 9.79 (s, 1H); 7.97 (d, *J* = 8.7 Hz, 2H); 7.72 (d, *J* = 8.7 Hz, 2H); 7.52 (d, *J* = 2.0 Hz, 1H); 7.42 (dd, *J* = 5.1, 1.1 Hz, 1H); 7.39 (dd, *J* = 8.3, 2.1 Hz, 1H); 7.32 (dd, *J* = 3.6, 1.0 Hz, 1H); 7.07 (dd, *J* = 5.1, 3.6 Hz, 1H); 6.93 (d, *J* = 8.3 Hz, 1H); 2.09 (s, 3H); ^13^C (150 mHz, DMSO- *d*_6_) δ 122.42, 128.73, 124,56, 144.0, 24.59, 169.36, 131.71, 128.8, 118.52, 129.30, 142.08, 165.50, 138.7, 124.19, 125.8 124.24, 119.0, 125.8; IR (KBr, cm^−1^) 3471.87, 1683.86, 1635.64, 1541.12, 1400.32, 1313.52; MS/ESI m/z calculated for C_19_H_16_N_2_O_2_S: 352.09, found: [M + H]+ 353.1; mp: 120–130 °C.

##### 4-Acetyl-*N*-(2-amino-5-(3,5-dimethylisoxazol-4-yl)phenyl)benzamide (**3**)

Light brown solid with 83% yield. **^1^**H NMR (600 mHz, DMSO- *d*_6_) δ 10.21 (s, 1H); 9.60 (s, 1H); 7.94 (d, *J* = 8.5 Hz, 2H); 7.70 (d, *J* = 8.7 Hz, 2H); 7.17 (s, 1H); 6.97 (dd, *J* = 8.2, 2.1 Hz, 1H); 6.86 (d, *J* = 8.2 Hz, 1H); 5.09 (s, 2H); 2.38 (s, 3H); 2.20 (s, 3H); 2.08 (s, 3H); ^13^C (150 mHz, DMSO- *d*_6_) δ 11.30, 164.00, 10.57, 158.21, 115.90, 24.17, 168.28, 128.69, 118.02, 128.76, 142.20, 164.85, 142.47, 127.03, 117.25, 126.88, 116.33, 123.52; IR (KBr, cm^−1^) 3446.79, 1683.86, 1635.64, 1608.62, 1541.12, 1506.41, 1404.18, 1363.67; MS/ESI m/z calculated for C_20_H_19_N_3_O_3:_ 365.13, found: [M + H]+ 366.12; mp: 182–185 °C.

#### 3.2.5. Procedures for the Synthesis of *N*-(2-Aminophenyl)-4-(3,5-dimethylisoxazol-4-yl)benzamide (**4**) 

##### Synthesis of 4-Methyl Iodine Benzoate (**30**)

In a 10 mL round-bottom flask, 1.6 mmol of 4-iodobenzoic acid (**29**), 4 mL of methanol, and 200 µL of sulfuric acid (3.5 mmol) were added. The reaction mixture was kept under stirring at 60 °C for 18 h. The reaction monitoring was carried out by TLC, using (9:1) hexane: ethyl acetate as the mobile phase. Next, the solvent was dried under reduced pressure, and the obtained solid was suspended using 150 mL of ethyl ether. The organic phase was washed three times using 30 mL of saturated sodium carbonate and three times using distilled water. The organic phase was dried with anhydrous sodium sulfate and filtered. The solvent was evaporated under reduced pressure leading to the formation of the desired product, 4-methyl iodine benzoate (**30**), as a white solid with 90% yield. **^1^**H NMR (600 mHz, DMSO- *d*_6_) δ 7.91 (m, 2H); 7.70 (m, 2H); 3.84 (s, 3H); ^13^C (150 mHz, DMSO- *d*_6_) δ 131.31, 102.28, 138.26, 129.54, 166.36, 52.80.

##### Synthesis of Methyl 4-(3,5-Dimethylisoxazol-4-yl)benzoate (**31**)

In a 10 mL round-bottom flask, 1.1 mmol of 4-methyl iodine benzoate (**30**), 1.5 mmol of 3,5-dimethyl-isoxazole boronic acid, 0.05 mmol of Pd(PPh_3_)_4_ (palladium tetrakis), 2.2 mmol of K_2_CO_3_ in 2 mL of water, and 5 mL of 1,4-dioxane were added. The Suzuki coupling reaction was carried out at 80 °C for 4 h. During this time, the reaction was kept under stirring and inert nitrogen atmosphere. The reaction was monitored by TLC, using hexane:ethyl acetate (1:1) as the mobile phase. Then, the reaction medium was filtered through celite to remove the palladium catalyst, washed with methanol (~100 mL) and rotary evaporated to eliminate the solvent leading to a brown solid. The crude was diluted with 150 mL of ethyl acetate and the organic phase was washed three times using 50 mL of distilled water. The organic phase was dried over anhydrous sodium sulfate and filtered. The solvent was evaporated under reduced pressure, and the compound was purified by column chromatography with petroleum ether/ethyl acetate (8:2) as eluents to provide the desired product, methyl 4-(3,5-dimethylisoxazol-4-yl)benzoate (**31**), at yields of 64%. **^1^**H NMR (600 mHz, DMSO- *d*_6_) δ 8.04 (m, 2H); 7.56 (m, 2H); 3.87 (s, 3H); 2.25 (s, 3H); 2.43 (s, 3H); ^13^C (150 mHz, DMSO-*d*_6_) δ 11.49, 165.99, 10.53, 158.03, 134.94, 138.26, 115.19, 131.31, 128.50, 165.97, 52.26.

##### Synthesis of 4-(3,5-Dimethylisoxazol-4-yl)benzoic Acid (**32**)

In a 10 mL round-bottom flask, 0.64 mmol of methyl 4-(3,5-dimethylisoxazol-4-yl) benzoate (**31**), 1.6 mmol of sodium hydroxide in 1 mL of water, and 4 mL of tetrahydrofuran were added. The reaction was kept under stirring at room temperature for 24 h. The reaction was monitored by TLC using hexane:ethyl acetate (8:2) as the mobile phase. Next, the solvent was dried, and the crude diluted with 150 mL of ethyl acetate. The organic phase was washed three times with citric acid 2M (30 mL), followed by three times of distilled water (30 mL). Subsequently, the organic phase was dried with anhydrous sodium sulfate and filtered. The solvent was evaporated under reduced pressure leading to the formation of the desired product, 4-(3,5-dimethylisoxazol-4-yl)benzoic acid (**32**), as a brown solid at a yield of 64%. **^1^**H NMR (600 mHz, DMSO- *d*_6_) δ 8.02 (m, 2H); 7.53 (m, 2H); 2.43 (s, 3H); 2.25 (s, 3H); ^13^C (150 mHz, DMSO- *d*_6_) δ 11.52, 165.93, 10.58, 158.11, 134.52, 129.00, 115.34, 129.82, 129.69, 167.08. 

##### *N*-(2-Aminophenyl)-4-(3,5-dimethylisoxazol-4-yl)benzamide (**4**)

A mixture containing 0.9 mmol of compound 4-(3,5-dimethylisoxazol-4-yl)benzoic acid (**32**) 1.1 mmol of *N*′-ethylcarbodiimide hydrochloride (EDC), 0.18 mmol of 4-(dimethylamino)pyridine (DMAP), and 5 mL of anhydrous *N*,*N*-dimethylformamide (DMF) was added in a 10 mL round-bottom flask at 0 °C. The reaction was stirred under a nitrogen atmosphere for 30 min. Next, 1 mmol of ortho-phenylenediamine (**33**) and 1 mmol of triethylamine were added dropwise at 0 °C. The reaction was kept under stirring at room temperature for 24 h. Reaction monitoring was carried out by TLC using ethyl acetate:hexane (6:4) as the mobile phase. Next, the medium was diluted with 150 mL of ethyl acetate and the organic phase was washed three times with 50 mL of distilled water. The organic phase was dried with anhydrous sodium sulfate and filtered. Then, the solvent was evaporated under reduced pressure and the purification was carried out by crystallization in dichloromethane to obtain *N*-(2-aminophenyl)-4-(3,5-dimethylisoxazol-4-yl)benzamide (**4**), a light purple solid at a yield of 15%. **^1^**H NMR (600 mHz, DMSO-*d*_6_) δ 9.74 (s, 1H); 8.07 (d, *J* = 8.2 Hz, 2H); 7.53 (d, *J* = 8.3 Hz, 2H); 7.17 (d, *J* = 7.3 Hz, 1H); 6.98 (m, 1H); 6.79 (dd, *J* = 8.0, 1.3 Hz, 1H); 6.61 (m, 1H); 4.91 (s, 1H); 2.44 (s, 3H); 2.26 (s, 3H); ^13^C (150 mHz, DMSO- *d*_6_) δ 10.61, 158.23, 11.52, 165.79, 115.52, 128.82, 133.07, 128.41, 133.61, 165.07, 143.32, 126.89, 126.73, 116.39, 116.22, 123.28; IR (KBr, cm^−1^) 3448.72, 1635.64, 1602.85, 1541.12, 1458.18, 1398.39, 1305.81; MS/ESI m/z calculated for C_18_H_17_N_3_O_2_: 308.11, found: [M + H]+ 309.12; mp: 240–247 °C.

#### 3.2.6. General Procedure for the Synthesis of Tert-butyl (2-(4-bromo-benzamide) Derivatives (**18**) and (**23**)

In a 10 mL round-bottom flask, 1.5 mmol of 4-bromo-benzoic acid (**17**) was mixed with 1.5 mmol of *N*′-ethylcarbodiimide hydrochloride (EDC), 0.3 mmol of 4-(dimethylamino)pyridine (DMAP), and 5 mL of anhydrous *N*,*N*-dimethylformamide (DMF). The reaction was stirred under a nitrogen atmosphere for 30 min. Then, 1.5 mmol of tert-butyl (2-amino-4-(thiophen-2-yl)phenyl)carbamate (**14**) or tert-butyl (2-amino-4-(3,5-dimethylisoxazol-4-yl)phenyl)carbamate (**21**) and 1.5 mmol of triethylamine was added dropwise, at 0 °C. The reaction was kept under stirring at room temperature for 24 h. Reaction monitoring was carried out by CCD using ethyl acetate:hexane elution as the mobile phase, with development under ultraviolet light (254 nm). Next, the reaction medium was diluted with 150 mL of ethyl acetate for liquid/liquid extraction. The organic phase was washed three times with 50 mL of distilled water. Subsequently, the organic phase was dried with anhydrous sodium sulfate and filtered. Then, the solvent was evaporated under reduced pressure, and, in the last step, the compounds were purified to provide the desired products (**18**) or (**23**).

##### Tert-butyl (2-(4-bromobenzamide)-4-(thiophen-2-yl)phenyl)carbamate (**18**)

Purification was carried out by crystallization in dichloromethane to obtain tert-butyl (2-(4-bromobenzamide)-4-(thiophen-2-yl)phenyl)carbamate (18) as a light brown solid with 25% yields. **^1^**H NMR (600 mHz, DMSO-d_6_) δ 9.99 (s, 1H); 8.74 (s, 1H); 7.94 (d, J = 8.5 Hz, 2H); 7.78 (m, 3H); 7.64 (d, J = 8.5 Hz, 1H); 7.45 (dd, J = 3.6, 1.0 Hz, 1H); 7.13 (dd, J = 5.1, 3.6 Hz, 1H); 1.45 (s, 9H); ^13^C (150 mHz, DMSO- d_6_) δ 79.73, 28.04, 153.22, 129.46, 123.97, 122.87, 131.60, 123.15, 129.59, 164.73, 133.39, 129.87, 131.46, 125.61, 142.62, 125.43, 128.51, 123.42.

##### Tert-butyl(2-(4-bromobenzamide)-4-(3,5-dimethylisoxazol)4yl)phenyl)carbamate (**23**)

Purification was carried out by crystallization in ethyl acetate to obtain tert-butyl(2-(4-bromobenzamide)-4-(3,5-dimethylisoxazol)4yl)phenyl)carbamate (**23**) as a brown solid with 29% yields. **^1^**H NMR (600 mHz, DMSO-*d*_6_) δ 9.94 (s, 1H); 8.83 (s, 1H); 7.91 (d, *J* = 8.0 Hz, 2H); 7.77 (d, *J* = 8.3 Hz, 2H); 7.66 (d, *J* = 8.4 Hz, 1H); 7.51 (s, 1H); 7.23 (d, *J* = 7.5 Hz, 1H); 2.42 (s, 3H); 2.24 (s, 3H); 1.45 (s, 9H); ^13^C (150 mHz, DMSO- *d*_6_) δ 79.87, 28.08, 153.42, 131.36, 123.91, 126.21, 125.32, 126.73, 129.63, 164.69, 133.47, 129.87, 131.56 125.68, 115.27, 165.15, 11.35, 158.16, 10.54.

#### 3.2.7. General Procedure for the Synthesis of Tert-butyl (2-(4-bromo-benzamide) Derivatives (**27**) and (**37**)

In a 10 mL round-bottom flask was added 1.5 mmol of bromobenzoic acid, 3 mmol of oxalyl chloride, two drops of dimethylformamide (DMF), and 5 mL of dichloromethane. The reaction mixture was maintained at room temperature for 1 h. Next, the reaction mixture was transferred to a 50 mL round-bottom flask and the solvent was evaporated under reduced pressure. In another 10 mL round-bottom flask, was added 1.5 mmol of tert-butyl (3-amino-[1,1’-biphenyl]-4-yl)carbamate (**26**) or 1.5 mmol of tert-butyl (2-amino-4-fluorophenyl)carbamate (**36**) and 3 mmol of methylmorpholine in 5 mL of dry THF. This content was maintained at 0 °C and was later added to the reaction medium at 0 °C. The reaction mixture was maintained at room temperature overnight. The reaction was monitored by TLC, using hexane:ethyl acetate (9:1) as the mobile phase. Then, the reaction medium was diluted with 150 mL of ethyl acetate for liquid/liquid extraction. The organic phase was washed three times with 50 mL of distilled water. Subsequently, the organic phase was dried with anhydrous sodium sulfate and filtered. Then, the solvent was evaporated under reduced pressure, and, in the last step, the compounds were purified to obtain the desired products (**27**) and (**37**).

##### Tert-butyl (3-(4-bromobenzamido)-[1,1’-biphenyl]-4-yl)carbamate (**27**)

Compound tert-butyl (3-(4-bromobenzamido)-[1,1’-biphenyl]-4-yl)carbamate (**27**) was purified by chromatography using the Biotage^®^ Isolera TM (SNAP ultra 10; with hexane/ethyl acetate 9:1 as eluents in an isocratic method; 36 mL/min flux and 254 nm wavelength detection), leading to the formation of the pure product, as a light brown solid at yield of 84%. **^1^**H NMR (600 mHz, DMSO-*d*_6_) δ 9.96 (s, 1H); 8.78 (s, 1H); 7.95 (d; *J* = 8.4 Hz; 2H); 7.77 (d; *J =* 8.4 Hz, 2H); 7.68 (m, 2H); 7.63 (m, 4H); 7.56 (m, 2H); 1.46 (s, 9H); ^13^C (150 mHz, DMSO- *d*_6_) δ 165.42, 163.04, 161.42, 153.78, 137.82, 135.12, 134.19, 132.52, 131.98, 131.91, 130.07, 129.27, 129.20, 128.82, 128.77, 124.80, 124.40, 116.30, 116.16, 80.22, 28.50.

##### Tert-butyl (4-fluoro-2-(4-iodobenzamido)phenyl)carbamate (**37**)

Compound tert-butyl (4-fluoro-2-(4-iodobenzamido)phenyl)carbamate (**37**) was purified by chromatography using the Biotage^®^ Isolera TM (SNAP ultra 10; with hexane/ethyl acetate (7:3) as eluents in an isocratic method; 36 mL/min flux and 254 nm wavelength detection), leading to the formation of the pure product, as a light brown solid at yield of 65%. **^1^**H NMR (600 mHz, DMSO-*d*_6_) δ 9.82 (s, 1H); 8.80 (s, 1H); 7.93 (d; *J* = 8.5 Hz; 2H); 7.74 (d; *J* = 8.4 Hz; 2H); 7.54 (dd; *J* = 11.2, 2.9 Hz; 1H); 7.41 (dd; *J* = 8.8, 6.3 Hz; 1H); 6.95 (ddd, *J =* 11.1, 8.4, 3.0 Hz, 1H); 1.44 (s, 9H). 

#### 3.2.8. General Procedure for the Synthesis of (**5**) (**6**), (**7**) and (**8**)

In a 10 mL round-bottom flask was added 0.4 mmol of tert-butyl (2-(4-bromobenzamide)-4-(thiophen-2-yl)phenyl)carbamate (**18**) or tert-butyl(2-(4-bromobenzamide)-4-(3,5-dimethylisoxazol)4yl)phenyl)carbamate (**23**) or tert-butyl (3-(4-bromobenzamido)-[1,1’-biphenyl]-4-yl)carbamate (**27**) or tert-butyl (4-fluoro-2-(4-iodobenzamido)phenyl)carbamate (**37**), 0.5 mmol of 3,5-dimethyl-isoxazole boronic acid, 0.02 mmol of Pd(PPh_3_)_4_ (palladium tetrakis), 0.8 mmol of K_2_CO_3_ in 2 mL of water, and 5 mL of 1,4-dioxane. The Suzuki coupling reaction was maintained at 80 °C for 4 h. During this time, the reaction was kept under stirring and an inert nitrogen atmosphere. The reaction was monitored by TLC, using hexane:ethyl acetate (1:1) as the mobile phase. Next, the reaction medium was filtered through celite to remove the palladium catalyst, washed with methanol (~100 mL), and rotary evaporated to eliminate the solvent. The crude was diluted with 150 mL of ethyl acetate and the organic phase was washed three times using 50 mL of distilled water. The organic phase was dried over anhydrous sodium sulfate and filtered. The solvent was evaporated under reduced pressure and the compounds were purified by column chromatography to obtain the Boc-protected intermediates (**19**), (**24**), (**28**), and (**38**) with 35% yields for all reactions. The last step consisted of the removal of the Boc-protecting group by adding, in a 10 mL round-bottom flask, about 0.2 mmol of the respective previous intermediate and trifluoroacetic acid (TFA) in excess (1 mmol). The reaction was maintained at room temperature and stirred for 3 h. The reaction was monitored by TLC, using ethyl acetate:hexane (1:1) elution as the mobile phase, developed under ultraviolet light (254 nm). Ninhydrin (2,2-dihydroxy-hydrindene-1,3-dione) solution at 5% was used for the detection of primary amines. TFA was removed under reduced pressure, and the solid obtained was suspended with 150 mL of ethyl acetate. The organic phase was washed three times with 50 mL of distilled water. The organic phase was dried over anhydrous sodium sulfate and filtered. Lastly, the solvent was evaporated under reduced pressure to obtain the desired products (**5**) (**6**), (**7**), and (**8**).

##### *N*-(2-Amino-5-fluorophenyl)-4-(3,5-dimethylisoxazol-4-yl)benzamide (**5**)

Brown solid with 64% yield. **^1^**H NMR (600 mHz, DMSO- *d*_6_) δ 9.65 (s, 1H); 8.07 (d; *J =* 8.2 Hz; 1H); 7.63 (m, 1H); 7.56 (m, 1H); 7.53 (d; *J* = 8.2 Hz; 1H); 6.55 (dd; *J* = 11.2, 2.8 Hz; 1H); 6.37 (td, *J =* 8.5, 2.8 Hz; 1H); 5.25 (s, 2H); 2.44 (s, 3H); 2.26 (s, 3H); IR (KBr, cm^−1^) 3273.20; 1639.49; 1539.20; 1423.47; 1290.38; MS/ESI m/z calculated for C_18_H_16_FN_3_O_2_: 326.13, found: [M+H]+ 327.12; mp: 98–102 °C.

##### *N*-(2-Amino-5-(thiophen-2-yl)phenyl)-4-(3,5-dimethylisoxazol-4-yl)benzamide (**6**)

Light brown solid with 85% yield. **^1^**H NMR (600 mHz, DMSO- *d*_6_) δ 9.80 (s, 1H); 8.10 (d, *J* = 7.6 Hz, 2H); 7.55 (d, *J* = 7.9 Hz, 2H); 7.47 (d, *J* = 1.8 Hz, 1H); 7.35 (dd, *J* = 5.1, 0.9 Hz, 1H); 7.31 (dd, *J* = 8.4, 2.1 Hz, 1H); 7.24 (dd, *J* = 3.5, 0.8 Hz, 1H); 7.05 (dd, *J* = 5.0, 3.6 Hz, 1H); 6.82 (d, *J* = 8.4 Hz, 1H); 5.19 (s, 2H); 2.45 (s, 3H); 2,28 (s, 3H); ^13^C (150 mHz, DMSO- *d*_6_) δ 121.03, 128.23, 123.24, 144.23, 10.52, 158.10, 11.44, 165.67, 115.42, 128.8, 128.37, 128.71, 133.06, 165.07, 143.12, 124.04, 123.07, 124.02, 116.33, 122.22; IR (KBr, cm^−1^) 3462.22, 1558.48, 1653.00, 1541.12, 1506.41, 1404.18, 1313,52; MS/ESI m/z calculated for C_22_H_19_N_3_O_2_S: 390.11, found: [M+H]+ 391.12; mp: 92–96 °C.

##### *N*-(2-Amino-5-(3,5-dimethylisoxazol-4-yl)phenyl)-4-(3,5-dimethylisoxazol-4-yl)benzamide (**7**)

Brown solid with 83% yield. **^1^**H NMR (600 mHz, DMSO-*d*_6_) δ 9.77 (s, 1H); 8.08 (d, *J* = 8.1 Hz, 2H); 7.55 (d, *J* = 8.2 Hz, 2H); 7.19 (m, 1H); 7.00 (dd, *J* = 8.2; 2.0 Hz, 1H); 6.87 (d, *J* = 8.2 Hz, 1H); 2.44 (s, 3H); 2.27 (s, 3H); ^13^C (150 mHz, DMSO- *d*_6_) δ 10.25, 158.22, 10.97, 164.02, 115.88, 10.24, 158.11, 11.08, 165.04, 115.43, 133.48, 128.56, 128.19, 133.07, 165.69, 142.60, 116.03, 117.17, 127.05, 127.00, 123.22; IR (KBr, cm^−1^) 3446.79, 1647.21, 1618.28, 1558.48, 1506.41, 1398.39, 1313.52; MS/ESI m/z calculated for C_23_H_22_N_4_O_3_: 403.17, found: [M+H]+ 404.17; mp: 85–88 °C.

##### *N*-(4-Amino-[1,1’-biphenyl]-3-yl)-4-(3,5-dimethyl-4,5-dihydroisoxazol-4-yl)benzamide (**8**)

White solid with 67% yield. **^1^**H NMR (600 mHz, Acetone-*d_6_*) δ 8.82 (s, 1H); 8.20 (d; *J* = 8.0 Hz; 1H); 8.06 (m, 1H); 7.72 (m, 2H); 7.59 (m, 4H); 7.50 (m, 1H); 7.43 (m, 1H); 7.39 (m, 2H); 2.86 (s, 2H); 2.48 (s, 3H); 2.31 (s, 3H). ^13^C (150 mHz, Acetone- *d_6_*) δ 165.69; 165.80; 158.00; 157;98; 140.81; 136.90; 129.29; 128.99; 128.84; 128.69; 128.20; 127.58; 127.07; 126.59; 126.13; 125.92; 124.99; 122.11; 120.20; 118.69; 10.79; 9.97; IR (KBr, cm^−1^) 3381.21, 1647.21, 1539.20, 1417.68, 1319.31; MS/ESI m/z calculated for C_24_H_23_N_3_O_2_: 386.18, found: [M+H]+ 387.19; mp: 162–166 °C. 

#### 3.2.9. General Procedure for the Synthesis of (**9**) and (**10**) 

In a 10 mL round-bottom flask, was added 1.1 mmol of 1-[bis(dimethylamino)methylene]-1H-1,2,3-triazolo[4,5-b]pyridinium 3-oxide hexafluorophosphate (HATU), 1.1 mmol of *N*-Ethyl-*N*-(propan-2-yl)propan-2-amine (DIPEA), 1 mmol of 4-(3,5-dimethylisoxazol-4-yl)benzoic acid (32), or 4-acetamidobenzoic acid (15) in 5 mL of DMF. The reaction was kept under constant stirring and a nitrogen atmosphere for 15 min. Next, about 1 mmol 4-fluoro-2-nitroaniline (34) was added and the reaction was maintained under stirring at room temperature for 24 h. For monitoring the reaction by TLC, hexane:ethyl acetate (1:1) was used as the mobile phase. Then, the medium was diluted with 150 mL of ethyl acetate and the organic phase was washed three times with 50 mL of distilled water. The organic phase was dried with anhydrous sodium sulfate and filtered. Then, the solvent was evaporated under reduced pressure and the compounds were purified by chromatography. 

##### 4-Acetamide-*N*-(4-fluoro-2-nitrophenyl)benzamide (**9**)

Compound 4-acetamide-*N*-(4-fluoro-2-nitrophenyl)benzamide (**9**) was purified by chromatography using the Biotage^®^ Isolera TM (SNAP ultra 10; with hexane/ethyl acetate 7:3 as eluents in an isocratic method; 36 mL/min flux and 254 nm wavelength detection), leading to formation of the pure product, as a white solid at yields of 55%. **^1^**H NMR (600 mHz, DMSO-*d*_6_) δ 10.30 (s, 1H); 8.75 (dd, *J* = 4.4, 1.4 Hz, 1H); 8.50 (dd, *J* = 8.4, 1.4 Hz, 1H); 7.87 (d, *J* = 8.7, 2H); 7.66 (d, *J* = 8.7, 2H); 7.51 (dd, *J =* 8.4, 4.4 Hz, 1H); 2.07 (s, 1H); ^13^C (150 mHz, DMSO- *d*_6_) δ 135.06, 151.65, 143.64, 121.33, 129.28, 124.90, 167.51, 139.64, 118.78, 130.84, 125.36, 169.72, 24.48; MS/ESI m/z calculated for C_15_H_12_FN_3_O_4_: 318.00, found: [M + H]+ 319.01; mp: 199–204 °C.

##### 4-(3,5-Dimethylisoxazol-4-yl)-*N*-(4-fluoro-2-nitrophenyl)benzamide (**10**)

Compound 4-(3,5-dimethylisoxazol-4-yl)--(4-fluoro-2-nitrophenyl)benzamide (**10**) was purified by chromatography using the Biotage^®^ Isolera TM (SNAP ultra 10; with hexane/ethyl acetate 6:4 as eluents in an isocratic method; 36 mL/min flux and 254 nm wavelength detection), leading to the formation of the pure product, as a brown solid at yields of 26%. **^1^**H NMR (600 mHz, DMSO-*d*_6_) 8.77 (dd, *J* = 4.4, 1.4 Hz, 1H); 8.54 (dd, *J* = 8.4, 1.4 Hz, 1H); 8.02 (m, 2H); 7.52 (m, 3H); 2.44 (s, 3H); 2.25 (s, 3H); ^13^C (150 mHz, DMSO- *d*_6_) δ 134.68, 151.48, 143.37, 121.31, 129.13, 124.90, 167.04, 134.49, 129.29, 130.10, 129.67, 115.31, 165.89, 11.49, 158.07, 10.55; MS/ESI m/z calculated for C_18_H_14_FN_3_O_4_: 356.10, found: [M+H]+ 357.11; mp: 134–140 °C.

### 3.3. Enzymatic Inhibition Assays

Compounds (**1**–**10**) were evaluated against HDAC-1, -2, -3, -4, -6, -11, and BRD4. All compounds were dissolved in DMSO and evaluated at a concentration of 10 μM. For HDAC assays, the non-selective inhibitor vorinostat (SAHA) was used as a control at a concentration of 0.5 μM. For BRD4, JQ1 was used as a control at a concentration of 2.5 μM. All assays were conducted in triplicate of three independent experiments and were expressed as the average of the results.

#### 3.3.1. HDAC Inhibition Assay

For the HDAC enzymatic assay, a mixture containing 50 μL of buffer, 5 μg of BSA (HDAC substrate; BPS Bioscience catalog number 50031) (BPS Bioscience, San Diego, CA, USA) respective HDAC enzyme (BPS Bioscience catalog number 50031) (10 μM), and 5 μL of each compound (**1**–**10**) (10 μM) to be evaluated were kept at 37 °C for 30 min. Vorinostat (SAHA; Cayman Chemicals (Ann Arbor, MI, USA Catalog Number 10009929) was used as a drug control. After enzymatic reactions, 2 × 50 μL of HDAC developer were added to each well for HDAC enzymes and the plate was incubated at room temperature for 15 min. The intensity of fluorescence was measured with an excitation of 360 nM and emission of 460 nM using a Tecan Infinite M1000 microplate reader. The fluorescence intensity data were analyzed using the GraphPad Prism software. In the absence of the compound to be tested, the intensity fluorescent (Ft) in each data set was defined as 100% activity. In the absence of HDAC, the fluorescent intensity (Fb) in each data set was set to 0% activity. The percentage of activity in the presence of each compound was calculated according to the following equation: % activity = (F − Fb)/(Ft − Fb), where F = the intensity of fluorescence in the presence of the compound. The % activity values versus a series of compound concentrations were plotted using non-linear regression analysis of the sigmoidal dose–response curve generated with the equation Y = B + (TB)/1 + 10 ((LogEC50-X) × Hill Slope), where Y = percentage of activity, B = minimum percentage of activity, T = percentage maximum activity, X = logarithm of the concentration for tested compounds and Hill Slope = slope factor or Hill’s coefficient.

#### 3.3.2. BRD4 Inhibition Assay

The assays with BRD4 were performed using TR-FRET (time-resolved fluorescence energy transfer) using recombinant bromodomain proteins (2.5 nM) (BPS Bioscience BRD4 (BD1 + BD2) TR-FRET Assay Kit, BPS Catalog #32612) and the BET ligand (32 nM). JQ-1 (Medchem express; US, catalog number HY-13030) was used as a reference drug. The TR-FRET signal obtained in the test was correlated with the amount of ligand conjugated to the bromodomain. The compounds were solubilized in DMSO and diluted (1:20) in the reaction buffer. To this solution, was added 2 μL and transferred to 20 μL of the reaction mixture (BRD4 (BD1 + BD2), inhibitor, BET ligand, and fluorescent markers) so that the final concentration of DMSO was 0.5% for all reactions. The reaction mixture was incubated for 120 min at room temperature, before reading the TR-FRET signal. The fluorescence of acceptor and donor markers was measured using a microplate reader Tecan Infinite M1000. The TR-FRET signal was obtained through the fluorescence ratio between accepting and donor markers. The inhibition test was carried out in duplicate for three separate experiments. The data were analyzed using the GraphPad Prism software. In wells that contained only the BET ligand, the signal TR-FRET (Ft) was considered as being 100% of activity. In wells where control (inhibitor) was greater than 100 times, the IC50 was used to determine the TRFRET signal (Fb) as 0% activity. The percentage of activity in the presence of each compound was calculated using the following equation: % activity = [(F − Fb)/(Ft − Fb)] × 100, where F = TR FRET signal in the presence of the compound. The percentage of inhibition was calculated from the following equation: % inhibition = 100 − % activity.

## 4. Conclusions

Epigenetic modifiers acting through HDAC class I inhibition and BRD4 binding were designed and synthesized. Chemical characterization was carried out by analytical methods such as NMR **^1^**H and ^13^C, mass spectrometry, and infrared spectroscopy. The ability of all compounds to interact with HDAC class I and BRD4 was investigated using molecular docking. The docking studies were consistent with inhibitory results. Compounds containing 4-thiophene and 4-phenyl at 2-amino-benzamide ring favor HDAC class I inhibition, while 3,5-dimethylisoxazole subunit favors BRD4 inhibition. All the compounds were more prone to inhibiting HDAC class I, mainly HDAC-1 and -2, than interacting with BRD4. For HDAC-1, the inhibitory effect ranged from 8 to 95% and for HDAC-2 these values ranged from 10 to 91%. The most active compounds (**3**) and (**5**) against BRD4, moderately reduced the activity by 25%. All designed compounds (**2**–**8**) exhibited effects against BRD4 and HDAC class I, mainly HDAC-1 and -2, being promising prototypes to be evaluated against viral infections by acting through epigenetic mechanisms.

## Figures and Tables

**Table 1:** Docking score of Glide (XP mode) for all compounds.

Compounds	Docking Score		
	HDAC-2	HDAC-3	BRD4
(**1**)	−8.451	−8.260	−2.956
(**2**)	−10.238	−8.490	−5.731
(**3**)	−6.940	−8.209	−4.839
(**4**)	−9.001	−8.608	−6.014
(**5**)	−9.003		−4.895
(**6**)	−10.101		−5.338
(**7**)	−10.036		−4.298
(**8**)	−11.101		−4.021
(**9**)	−8.567		−4.975
(**10**)	−8.459	−7.548	−4.320

**Table 2:** Percentage inhibition effects induced by compounds (**1**–**10**) when evaluated against HDAC-1, -2, -3, -4, and -11, and BRD4 at concentration of 10 µM.

Compounds	Inhibitory Activity (%)					
	HDAC-1	HDAC-2	HDAC-3	HDAC-4	HDAC-11	BRD4
(**1**)	89	82	77	-	-	0
(**2**)	95	91	18	<5	<5	14
(**3**)	8	10	<5	<5	<5	25
(**4**)	75	72	57	<5	<5	21
(**5**)	11	10	23	<5	<5	20
(**6**)	95	88	16	<5	<5	17
(**7**)	8	10	<5	<5	<5	25
(**8**)	92	76	7	<5	<5	12
(**9**)	9	<5	<5	<5	<5	9
(**10**)	15	8	<5	<5	<5	5
Vorinostat ^1^	90	89	90	-	-	0
JQ-1 ^2^	-	-	-	-	-	88

^1^ Vorinostat (SAHA), a pan-HDAC inhibitor, was tested at 0.5 µM. ^2.^JQ-11 at a concentration of 2.5 µM was used as a standard against BRD4. (-): not determined.

## Data Availability

Data is contained within the article and Supplementary Material.

## Supplementary Materials

The following are available online at https://www.mdpi.com/article/10.3390/ph14121308/s1, Table S1: In silico physico-chemical and ADME properties obtained using the SwissADME tool.
