# Supplementary material for: Design and Synthesis of Hybrid Compounds as Epigenetic Modifiers
Source: Pharmaceuticals (Basel). 2021 Dec 15;14(12):1308. doi: 10.3390/ph14121308 (PMC8709175; doi:10.3390/ph14121308)
Supplement: Supplementary file 1 [file pharmaceuticals-14-01308-s001.zip › pharmaceuticals-1486035-supplementary.pdf]

Physico-chemical properties	Calculated values									
	**1**	**2**	**3**	**4**	**5**	**6**	**7**	**8**	**9**	**10**
Num. heavy atoms	20	25	27	23	24	28	30	29	26	23
Num. arom. heavy atoms	12	17	17	17	17	22	22	23	17	12
Fraction Csp^3	0.07	0.05	0.15	0.11	0.11	0.09	0.17	0.08	0.11	0.07
Num. rotatable bonds	5	6	6	4	4	5	5	5	5	6
Num. H bond receptors	2	2	4	3	4	3	5	3	6	5
Num. H bond donors	3	3	3	2	2	2	2	2	1	2
Log $P_{o/w}$ (iLOGP)	1.70	2.21	2.49	2.46	2.50	3.26	3.23	3.08	2.42	2.03
Water solubility (mg/mL)(ESOL)	1.04	6.14 e-2	3.05 e-2	3.99 e-2	2.98e-2	2.41 e-3	4.47 e-3	1.68 e-3	5.81 e-3	2.44 e-1
class	Soluble	Moderately soluble	Moderately soluble	Soluble	Moderately soluble	Moderately soluble	Moderately soluble	Moderately soluble	Moderately soluble	Soluble
GI absorption	High	High	High	High	High	Low	High	High	High	High
BBB permeant	No	No	No	No	No	No	No	No	No	No
P-gp substrate	No	Yes	Yes	Yes	Yes	Yes	Yes	Yes	No	No
CYP1A2 inhibitor	No	Yes	Yes	Yes	Yes	Yes	No	Yes	Yes	No
CYP2C19 inhibitor	No	Yes	No	Yes	No	Yes	Yes	Yes	Yes	No
CYP2C9 inhibitor	No	Yes	Yes	No	No	Yes	Yes	Yes	Yes	Yes
CYP2D6 inhibitor	No	Yes	Yes	Yes	Yes	Yes	Yes	Yes	No	No
CYP3A4 inhibitor	No	Yes	Yes	Yes	Yes	Yes	Yes	Yes	Yes	Yes
Log *kp* (skin permeation)	-7.06 cm/s	-6.60 cm/s	-7.01 cm/s	-6.08 cm/s	-6.12 cm/s	-5.63 cm/s	-6.04 cm/s	-5.39 cm/s	-5.55 cm/s	-6.75 cm/s
Lipinski	Yes;	Yes	Yes	Yes	Yes	Yes	Yes	Yes	Yes	Yes
Ghose	Yes	Yes	Yes	Yes	Yes	Yes	Yes	Yes	Yes	Yes
Veber	Yes	Yes	Yes	Yes	Yes	Yes	Yes	Yes	Yes	Yes
Egan	Yes	Yes	Yes	Yes	Yes	Yes	Yes	Yes	Yes	Yes
Muegge	Yes	Yes	Yes	Yes	Yes	Yes	Yes	Yes	Yes	Yes

Bioavailability Score	0.55	0.55	0.55	0.55	0.55	0.55	0.55	0.56	0.55	0.55
PAINS alert	0	0	0	0	0	0	0	0	1 (nitro)	1 (nitro)
Synthetic accessibility	1.62	2.65	3.14	2.83	2.86	3.28	3.34	3.25	3.07	2.42

Pharmacokinetics: High and Low indicates gastrointestinal absorption; Yes and No indicates BBB (Blood Brain Barrier) permeation or P-gp (glycoprotein P). **Druglikeness:** Yes and No indicates that the molecule is according to all Lipinski, Ghose, Veber, Egan or Muegge descritors. **Bioavailability Score**: Predict the probability of a compound to have at least 10% oral bioavailability in rat or measurable Caco-2 permeability.
